# Clover: a clustering-oriented de novo assembler for Illumina sequences

**DOI:** 10.1186/s12859-020-03788-9

**Published:** 2020-11-17

**Authors:** Ming-Feng Hsieh, Chin Lung Lu, Chuan Yi Tang

**Affiliations:** 1 Department of Computer Science, National Tsing Hua University, Hsinchu, 30013 Taiwan; 2 Department of Computer Science and Information Engineering, Providence University, Taichung, 43301 Taiwan

**Keywords:** De novo genome assembly, DNA sequencing, De bruijn graph

## Abstract

**Background:**

Next-generation sequencing technologies revolutionized genomics by producing high-throughput reads at low cost, and this progress has prompted the recent development of de novo assemblers. Multiple assembly methods based on de Bruijn graph have been shown to be efficient for Illumina reads. However, the sequencing errors generated by the sequencer complicate analysis of de novo assembly and influence the quality of downstream genomic researches.

**Results:**

In this paper, we develop a de Bruijn assembler, called Clover (clustering-oriented de novo assembler), that utilizes a novel *k*-mer clustering approach from the overlap-layout-consensus concept to deal with the sequencing errors generated by the Illumina platform. We further evaluate Clover’s performance against several de Bruijn graph assemblers (ABySS, SOAPdenovo, SPAdes and Velvet), overlap-layout-consensus assemblers (Bambus2, CABOG and MSR-CA) and string graph assembler (SGA) on three datasets (*Staphylococcus aureus*, *Rhodobacter sphaeroides* and human chromosome 14). The results show that Clover achieves a superior assembly quality in terms of corrected N50 and E-size while remaining a significantly competitive in run time except SOAPdenovo. In addition, Clover was involved in the sequencing projects of bacterial genomes *Acinetobacter baumannii* TYTH-1 and *Morganella morganii* KT.

**Conclusions:**

The marvel clustering-based approach of Clover that integrates the flexibility of the overlap-layout-consensus approach and the efficiency of the de Bruijn graph method has high potential on de novo assembly. Now, Clover is freely available as open source software from https://oz.nthu.edu.tw/~d9562563/src.html.

## Background

Massively parallel DNA sequencing has become a prominent tool in biological research [1, 2]. The high-throughput and low cost of next-generation sequencing technologies produce high coverage of reads. The Illumina platform is one of the most commonly used sequencers, producing reads with lengths ranging from 35 to 300 bp. The de Bruijn graph approach is prevalent in the de novo assembly using Illumina reads, and it constitutes all possible substrings of length *k* (termed *k*-mers) from the reads to efficiently process the huge sequencing data. Choosing the length of *k* is an important issue in the de Bruijn graph approach. Theoretically, for reads without sequence errors, smaller *k*-mers increase the connectivity of the graph and larger *k*-mers decrease the number of ambiguous repeats in the graph. There is therefore a balance between sensitivity and specificity determined by *k* [3]. However, for reads with errors, larger *k*-mers decrease the sensitivity and specificity further due to sequencing errors generated by the Illumina platform, in which the primary errors are substitution errors, at rates of 0.5–2.5% [4].

In this study, we are trying to answer what happens if we design an approach to allow such errors on *k*-mers, and could the error allowance on *k*-mers improve the quality of de novo assembly. Therefore we developed a clustering-oriented approach, called Clover, to deal with those substitution errors, and use a new parameter *p* which describes the level of error allowance on *k*-mers. For example, setting *k* to 40 and *p* to 1 means that our algorithm uses each 40-mers in the input reads while allowing each of them to have the flexibility of 1 substitution error.

With the flexibility of error allowance on *k*-mers, Clover tries to cluster these *k*-mers together when their Hamming distance less than or equal to *p*, and merges each cluster of *k*-mers to a node by finding its consensus sequence. To avoid over-merging of clusters, which may occur on the boundary of repeat sequence, Clover will split each node into multiple nodes when the merged node has multiple major consensus sequences (see Implementation section for detail).

After the steps mentioned above, the number of nodes in the graph will dramatically reduce, which therefore simplifies analysis of assembly. For example, Table 1 compares three results of a *Leptospira shermani* assembly when using different level of error allowance (*p* = 0, 1 and 2). Setting *p* to 0 is equal to run our assembler with traditional de Bruijn-based approach, which does not have the flexibility of error allowance on *k*-mers. The assembly result shows that only setting *p* to 1 could dramatically increase the N50 both in contig and scaffold because it reduces the number of nodes to build the de Bruijn graph that increases the specificity. The result also shows that setting *p* to 2 only increases the N50 on contig whereas decreases on scaffold. In this case, reducing too many nodes could increase the specificity, but it seems losing some meaningful information, which decreases the sensitivity at the same time.

**Table 1:** The clustering effect of Clover on *Leptospira shermani* assemblies

p	Nodes	Contigs				Scaffolds				Time (min)	Memory (GB)
		Num	Total (kb)	Max (kb)	N50 (kb)	Num	Total (kb)	Max (kb)	N50 (kb)		
0	33,692,986	5070	3818	9	1.0	182	3947	178	54	45.8	17.7
1	15,969,082	1201	3875	34	5.5	117	3897	196	85	30.5	16.2
2	11,917,810	1103	3859	27	6.1	117	3880	145	63	36.0	11.7

*p* the level of error allowance on the *k*-mers, *Nodes* the number of nodes to build de Bruijn graph, *Num* the number of sequences produced, *Total* the total length of sequences produced, *Max* the maximum length of sequences produced, *N50* the N50 statistic calculated with respect to the total length of sequences produced, *Time* the run time to assemble the genome, *Memory* the memory requirement to assemble the genome

The memory requirements, as shown in Table 1, are in clear proportion to the number of nodes to build the de Bruijn graph, but are not in obvious proportion to parameter *p* (Additional file 4: Table S1, compares the memory requirements when using different *k* and *p*). The time cost is dropped when setting *p* to 1 due to the benefit of simplifying analysis, but setting *p* to 2 could not get the benefit more. Together with the phenomena described above, we should choose a suitable *p*, not as large as possible. In the case of *L. shermani* assembly, the suitable *p* is 1 and *p*/*k* is 2.5%, which is nearly the error rate of Illumina platform.

## Implementation

**Fig. 1:**
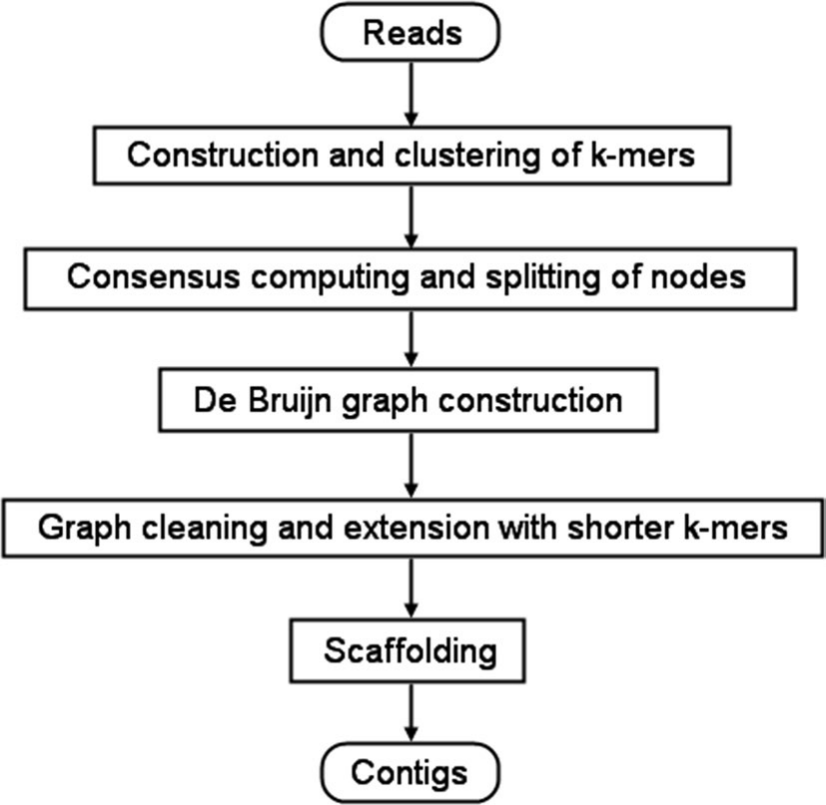
The flowchart of the Clover pipeline

Clover proceeds through the following phases whose flowchart is shown in Fig. 1.

### Construction and clustering of *k*-mers

**Fig. 2:**
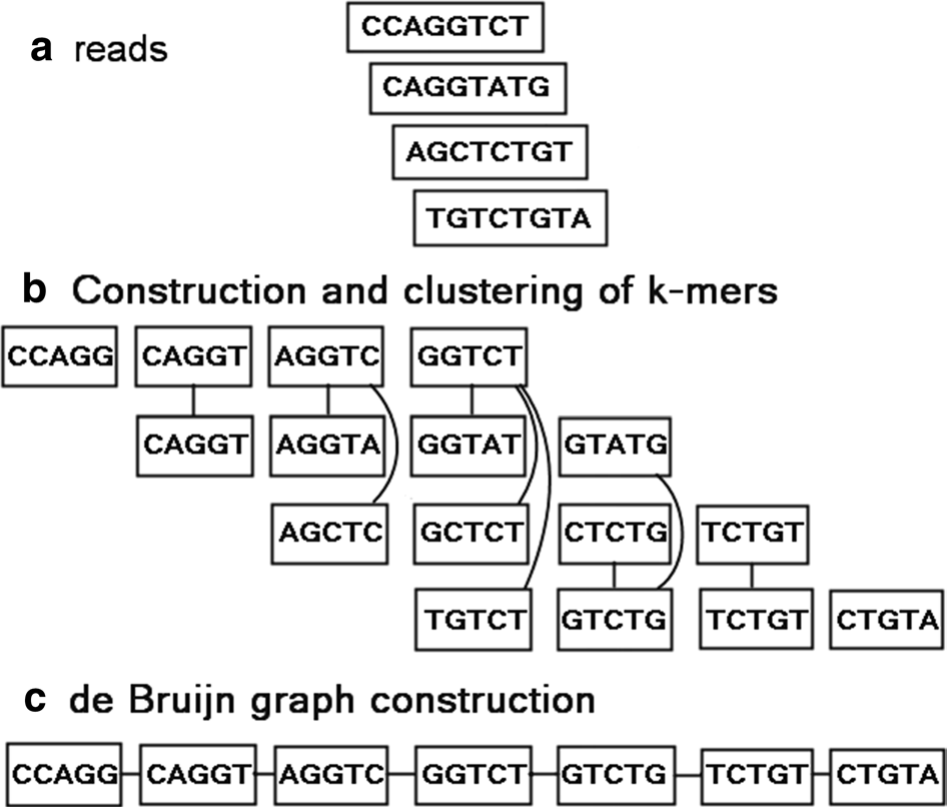
An example of 5-mers clustering while allowing 1 error

For given *k* (*k*-mer) and *p* (error allowance), Clover constructs a Hamming graph by extracting all the input *k*-mers as nodes. The graph’s edges are created by the pairs of *k*-mers if the Hamming distance of the *k*-mers (or their reverse complements) is ≤ *p*. Figure 2 illustrates an example of 5-mers clustering while allowing 1 error. Basically, the components of the Hamming graph are the clusters of *k*-mers (Fig. 2b). Clover then merges all the nodes within each component of the Hamming graph into a single node and computes its consensus sequence. In practice, just setting *p* to (*k* × error rate of sequencer) can dramatically reduce the number of *k*-mers for constructing the de Bruijn graph later and accelerates the subsequent graph processing. In the implementation, Clover uses two steps to cluster the *k*-mers: Step 1 extracts all *k*-mers from the input reads. Step 2 constructs a Hamming graph of the *k*-mers and then performs a breadth-first search to find each component in the Hamming graph. If there is no error allowance needed (*p* = 0), Clover will omit the process of step 2.

### Consensus computing and splitting of nodes

Clover computes the consensus sequence for each merged node by selecting the nucleotide with the most occurrence on each base pair. For example, the fourth component of Fig. 2b has 4 *k*-mers (GGTCT, GGTAT, GCTCT and TGTCT) whose consensus sequence therefore is GGTCT as shown in Fig. 2c. To avoid over-merging, if the *k*-mers of the merged node, called *v*, have a nucleotide on a base pair, called *x*, whose occurrence is closed to that of the corresponding nucleotide, called *y*, in the consensus sequence, Clover splits the merged node into multiple nodes. More realistically, given a fractional threshold *sp*, if the occurrence of *x* is greater than or equal to *sp* times the occurrence of *y*, Clover collects all the *k*-mers with *x* into a new node, called *v*_1_, leaves the others into a new node, called *v*_2_, and recursively retries this splitting process on *v*_1_ and *v*_2_. Let *q* be the number of *k*-mers in the merged node. Then the consensus computing requires *O*(*q* × *k*) time, the condition checking requires *O*(*k*) time and collecting the *k*-mers into the new nodes requires *O*(*q*) time. Therefore, similar to the analysis of quick sort, the worst case of the time complexity on the whole process is *O*(*q*^2^ × *k*). Figure 2c shows the resulting consensus sequences of Fig. 2b by setting *sp* to 0.6. Finally, Clover collects all the resulting consensus sequences as the *k*-mer set for constructing de Bruijn graph in the next phase.

### De Bruijn graph construction

For the *k*-mer set obtained in the previous phase, Clover constructs the de Bruijn graph by directly using the *k*-mer set as its node set. For any two nodes, it creates an edge between them if their corresponding *k*-mers have an overlapping of length *k* − 1. Figure 2c provides an example for de Bruijn graph construction.

### Graph cleaning and extension with shorter *k*-mers

Clover provides multiple operations based on spectrum, structure and their combination for removing spurious edges from the de Bruijn graph. The spectral error removal operation is the trimming of low-frequency edges. The structural error removal operations are the pruning of tips, bubbles, and erroneous connections. If there are multiple options during pruning of tips and bubbles, Clover prunes the low-frequency edges first. All these operations were also used in SOAPdenovo [5] and Velvet [3]. Clover then iteratively extends the graph by connecting two paths if the sequences of the paths have an overlapping length shorter than *k* until the given minimum overlapping length *m* is achieved. Clover defaultly sets the parameter *m* to (*k*/2) + 1. For example, we compare the different settings of *m* on the *Rhodobacter sphaeroides* assemblies (see Additional file 4: Table S2), where *k* is set to 46 in this case, and find that the default value 24 of *m* has the best scaffold result.

### Scaffolding

Clover utilizes read-pair information by aligning both ends of the pairs to the paths in the graph to find pairs of anchors. Given a scaffold support *ss*, which defaultly is set to 5, Clover links each pair of paths with the consistent bound if its support from the pairs of anchors is greater than or equal to *ss*. Clover calculates the medium length of insert sizes inferred from the pairs of anchors to be the consistent bound of the linked pair of paths. Finally, Clover predicts contigs by searching Eulerian super-paths on the graph.

## Results and discussion

To evaluate the assembly correctness of Clover, we have tested three typical datasets in the GAGE study: *Staphylococcus aureus* (2.9 Mb), *Rhodobacter sphaeroides* (4.6 Mb) and human chromosome 14 (88.3 Mb) [6]. Each dataset has original reads, Quake corrected reads and Allpaths-LG corrected reads. The result with the best scaffold N50 on these three datasets is selected for assembly comparison we will discuss later.

### Running Clover assembler

We provide Clover source code with this submission (see Additional file 1) and at our website https://oz.nthu.edu.tw/~d9562563/src.html.

Installation of Clover is provided at Additional file 2.

Each dataset in the GAGE study (see Additional file 2) is available at https://gage.cbcb.umd.edu/data/.

For testing Clover, test data is available at ‘Test Case’ of our website.

In Table 2, the results of our Clover were obtained from Allpaths-LG corrected reads. The assembly instructions used by our Clover are:

- *S. aureus*: clover -k 32 -p 0 -i1 frag_1.fastq,shortjump_1.fastq -i2 frag_2.fastq,shortjump_2.fastq -cs 5 -ss 3 -is 180,3500 -hp 0.6 -pm -ml 700.
- *R. sphaeroides*: clover -k 46 -p 0 -i1 frag_1.fastq,shortjump_1.fastq -i2 frag_2.fastq,shortjump_2.fastq -cs 7 -ss 3 -is 180,3500 -hp 0.6 -pm -ml 200.
- Human chromosome 14: clover -k 80 -p 3 -i1 frag_1.fastq,shortjump_1.fastq,longjump_1.fastq -i2 frag_2.fastq,shortjump_2.fastq,longjump_2.fastq -cs 9 -ss 5 -is 155,2543,35306 -hp 0.8 -ml 900.

**Table 2:** Comparison of assemblers on *Staphylococcus aureus* (SA), *Rhodobacter sphaeroides* (RS) and human chromosome 14 (HG)

Data (Mb	Assembler	Contigs						Scaffolds					
		Num	N50 (kb)	E-size (kb)	Errs	N50C (kb)	E-sizeC (kb)	Num	N50 (kb)	E-size (kb)	Errs	N50C (kb)	E-sizeC (kb)
SA	Clover	128	43.9	53.1	13	41.3	50.5	**12**	1490	947	2	**1490**	890
2.9	ABySS	**90**	129.1	181.1	16	**69.8**	**102.5**	61	170	199	**0**	107	127
	Bambus2	109	50.2	69.1	178	16.7	19.5	17	1084	1120	**0**	1084	**1120**
	CABOG	Could not run because of incompatible read lengths in one library											
	MSR-CA	94	59.2	60.4	22	49.2	51.4	17	**2412**	**2026**	1	1022	1039
	SGA	1252	4.0	4.7	**3**	4.0	4.7	546	208	166	2	208	164
	SOAPdenovo	107	**288.2**	**252.3**	58	62.7	67.5	99	332	302	**0**	288	227
	SPAdes	98	62.6	87.9	9	57.0	75.1	41	1703	1144	2	684	570
	Velvet	162	48.4	60.3	19	41.5	49.8	45	762	664	18	284	282
RS	Clover	453	20.1	23.8	19	**19.5**	**21.9**	59	2483	1795	1	2483	1795
4.6	ABySS	644	19.7	25.1	57	13.3	18.5	414	51	56	**0**	46	47
	Bambus2	**177**	93.2	94.5	360	12.8	16.3	92	2439	1375	1	390	1106
	CABOG	322	20.2	24.1	31	17.9	21.5	130	66	520	3	65	381
	MSR-CA	395	22.1	24.2	32	19.1	21.5	**43**	**2976**	**2039**	3	**2976**	**2017**
	SGA	3067	2.3	3.3	**4**	2.3	3.3	2096	51	53	**0**	51	53
	SOAPdenovo	204	**131.7**	**157.2**	401	14.6	18.7	166	660	688	**0**	660	559
	SPAdes	768	11.8	13.7	7	11.7	13.5	352	718	840	**0**	718	840
	Velvet	583	15.7	18.6	24	14.5	16.9	178	353	380	16	301	352
HG	Clover	24,527	3.4	5.3	718	3.2	5.0	2089	839	943	385	**409**	**502**
88.3	ABySS	21,222	14.7	19.0	1876	10.4	13.4	19,249	18	24	**13**	13	19
	Bambus2	13,592	5.9	23.3	8175	4.3	6.3	1792	324	528	240	200	274
	CABOG	**3361**	**45.3**	**58.8**	2346	**23.7**	**30.6**	**479**	393	549	39	309	457
	MSR-CA	30,103	4.9	6.8	1656	4.3	5.9	1425	893	1420	1430	282	407
	SGA	56,939	2.7	3.8	**375**	2.7	3.7	30,975	83	113	24	81	111
	SOAPdenovo	21,818	16.7	21.9	6587	7.8	10.4	13,502	454	533	384	227	276
	SPAdes	16,854	12.7	16.7	1519	10.4	13.6	9245	173	223	199	129	162
	Velvet	45,564	2.3	3.3	3665	2.1	3.0	3565	**1190**	**1825**	8659	86	124

*Num* the number of sequences produced, *N50* the N50 statistic calculated with respect to the genome size, *E-size* the most likely size of the sequence containing some random base in the genome, *Errs* the number of misjoins and for the contig value, also the number of indels > 5 bases, *N50C* the N50 calculated after splitting all sequences at error locations, and *E-sizeC* the E-size calculated after splitting all sequences at error locations. The best result in each column, for each dataset, is indicated in bold

The assembly results display all statistics data in the screen (see Additional file 3: Section S2) and create two assembly output files named out_contig.fasta and out_scaffold.fasta.

### Running *Leptospira shermani* assembly

We provide *Leptospira shermani* dataset tar file at our website https://oz.nthu.edu.tw/~d9562563/src.html.

Download and unpack it: tar -zxvf leptospirashermanidata.tar.gz.

The assembly instructions are: clover -k 40 -p ? -is 485 -i1 Lepto_500_1.fq -i2 Lepto_500_2.fq -hp 0.6, where ? runs with 0, 1 and 2, respectively.

The assembly results display all statistics data in the screen (see Additional file 3: Section S1) and create two assembly output files named out_contig.fasta and out_scaffold.fasta.

### Assemblers

Table 2 shows the comparison of Clover’s performance against several modern assemblers, which include ABySS [7], Bambus2 [8], CABOG [9], MSR-CA [10], SGA [11], SOAPdenovo [5], SPAdes [12] and Velvet [3]. All assembly statistics were generated by the GAGE validation scripts. Bambus2, CABOG and MSR-CA are well known overlap-layout-consensus assemblers, while ABySS, SOAPdenovo, SPAdes and Velvet are famous de Bruijn graph assemblers and SGA is typical string graph assembler.

### Comparison

For the *S. aureus* dataset, SOAPdenovo and ABySS have produced the longest two contigs. SGA, SPAdes, Clover and ABySS have been detected the fewest four errors in contigs, but SOAPdenovo contains many errors. Therefore ABySS achieves the longest corrected contigs. MSR-CA has produced the longest scaffolds, but its longest scaffold has been broken by an error. Instead, Clover and Bambus2 achieve the longest corrected scaffolds in terms of N50 and E-size respectively.

For the *R. sphaeroides* dataset, SOAPdenovo and Bambus2 have produced the longest two contigs. However, considering the assembly correctness, Clover achieves the longest corrected contigs. SGA and SPAdes contain fewest two errors in contigs, but their N50 lengths are relatively shorter. Excluding SGA and SPAdes, Clover’s contigs contain fewest errors. On the other hand, MSR-CA and Clover have the best two scaffold results both in uncorrected and corrected N50.

For the human chromosome 14 dataset, Clover produces the relatively conservative contigs, but its contigs contain fewest errors except SGA. CABOG has the best contig results both in uncorrected and corrected N50. Velvet produces the longest scaffolds. However, when focusing on the assembly correctness, Clover achieves the longest corrected scaffolds.

Note that the N50 statistics is defined as the minimum contig length (in descending order) needed to cover 50% of the genome. The N50 statistics generated by Clover is the minimum contig length needed to cover 50% of all the sequence produced. However, the N50 statistics generated by the GAGE validation scripts is the minimum contig length needed to cover 50% of the reference genomic sequence provided in GAGE study. Therefore the N50 statistics of Clover in Additional file 3 and the N50 statistics of Clover in Table 2 have a little difference.

The result of *L. shermani* seems to be poorer than *S. aureus* and *R. sphaeroides*. However, these two datasets in GAGE study have two libraries and the fragment size is up to 3500 bp [6], whereas the *L. shermani* dataset only has single library with fragment size 485 bp. The better assembly quality is caused by using more libraries. Practically, researchers usually use optical mapping to arrange scaffolds and then obtain the draft sequence [12].

In addition, our clustering approach can apply on ever error-corrected reads. For example, the assembly of human chromosome 14 is generated by clustering 80-mers while allowing 3 errors on Allpaths-LG corrected reads. Therefore Clover would not conflict with current error correction tools. In practice, we will apply smaller *k*-mer on single library or lower coverage dataset such as the *L. shermani* assembly, and larger *k*-mer on more complex genomes such as human chromosome 14. When using large *k*-mer, increasing the level of error allowance is especially needed even on ever error-corrected reads.

### Run times and memory requirements

To assess Clover’s run times and memory requirements, we have rerun above assemblers that follow the same processes and parameters of GAGE with their newest version on a 16-core AMD Opteron 6128 2 GHz server with 256 GB of RAM. The parameters of optimal result seem varying with the different version of assemblers and hence we only take their run times and memory requirements into comparisons. Because we don’t have large-scale parallel environment, we only run ABySS on single-process version.

Table 3 shows the comparison of these assemblers on run times and memory requirements. The result shows that the run time of Clover is significantly competitive to those efficient de Bruijn graph assemblers except SOAPdenovo. SOAPdenovo is the fastest assembler due to the multi-process parallelization.

**Table 3:** Comparison of assemblers on run times and memory requirements

Assembler	*Staphylococcus aureus*		*Rhodobacter sphaeroides*		Human Chromosome 14	
	Time	Memory	Time	Memory	Time	Memory
Clover	5.6 min	10.1 GB	13.9 min	11.0 GB	10.4 h	59.3 GB
ABySS	5.1 min	0.5 GB	11.6 min	0.5 GB	6.7 h	3.3 GB
Bambus2	55.5 min	2.3 GB	3.7 h	12.3 GB	5.1 d	190.3 GB
CABOG	NA*	NA*	2.9 h	12.3 GB	22.9 h	190.4 GB
MSR-CA	25.5 min	26.2 GB	41.3 min	28.3 GB	1.3 d	34.6 GB
SGA	35.5 min	1.1 GB	1.1 h	3.5 GB	18.8 h	35.0 GB
SOAPdenovo	2.3 min	3.1 GB	1.8 min	5.0 GB	2.1 h	8.0 GB
SPAdes	56.8 min	6.1 GB	29.5 min	4.5 GB	10.9 h	22.0 GB
Velvet	5.4 min	0.4 GB	7.3 min	0.5 GB	11.7 h	72.3 GB

*Time* the run time to assemble the genome, *Memory* the memory requirement to assemble the genome

*NA, could not run because of incompatible read lengths in one library

The major cost of Clover is the *k*-mers clustering. In the *k*-mers clustering, Clover constructs a Hamming graph in which it links each pair of *k*-mers as an edge if the Hamming distance of the pair of *k*-mers is ≤ *p*. To accelerate the process, Clover utilizes the indexing technique that partitions a *k*-mer into (*p* + 1) substrings. If the Hamming distance of a pair of *k*-mers is ≤ *p*, there must exist a pair of substrings that are exactly the same. Therefore Clover uses (*p* + 1) hash tables which index each substring of all *k*-mers to find the candidate pairs of *k*-mers, and performs comparisons to check their real Hamming distances. Let *n* be the number of the reads. *l* be the length of the reads, *k* be the length of *k*-mers and *p* be the level of error allowance on the *k*-mers. Note that *p* can be 0 in this study. Then *n* × (*l* − *k* + 1) is the number of *k*-mers within the reads, (*p* + 1) is the number of hash tables needed to find the candidate pairs of *k*-mers and each comparison for them requires *O*(*k*) time. Therefore, the worst case of the time complexity on *k*-mers clustering is *O*(*n* × (*l* − *k* + 1) × *k* × (*p* + 1)) ≅ *O*(*n* × (*l* − *k*) × *k* × (*p* + 1)). Similarly, the memory needed to store all the sequences of the *k*-mers is *O*(*n* × (*l* − *k*) × *k*), and the memory needed for all the *k*-mers on the hash tables is *O*(*n* × (*l* − *k*) × (*p* + 1)). Since *k* is much larger than *p*, the worst case of the space complexity on *k*-mers clustering is *O*(*n* × (*l* − *k*) × *k*).

### Sequencing projects

It is worth mentioning that Clover was involved in two sequencing projects to respectively sequence bacterial genomes *Acinetobacter baumannii* TYTH-1 (4.0 Mb and 165 contigs) [14] and *Morganella morganii* KT (3.8 Mb and 58 contigs) [15]. The contigs generated by Clover were then used to build the draft sequences, which were confirmed by optical mapping and PCR. From the draft sequences of *A. baumannii* TYTH-1 and *M. morganii* KT, 3682 and 3565 protein-coding sequences, 75 and 72 tRNA genes, and 6 and 10 rRNA genes were further predicted, respectively. Table 4 shows the summary of these two sequencing results.

**Table 4:** The results of two bacterial genome sequencing projects

	*Acinetobacter baumannii* TYTH-1	*Morganella morganii* KT
Length of sequence	3,957,368 bp	3,826,919 bp
Number of contigs	165	58
GC content	39%	51%
Number of protein-coding sequences	3682	3565
Number of tRNA genes	75	72
Number of rRNA genes	6	10
GenBank accession number	CP003856	ALJX00000000

The NGS datasets of these two bacterial sequencing projects are available for download at https://oz.nthu.edu.tw/~d9562563/src.html

### Limitations

The limit of our current Clover is that it cannot apply on genomes with large size up to 250 Mb. This is caused by 256 GB of RAM in our server (see Run times and memory requirements section for detail). However, if the server has more RAM, the limitation could be eliminated. The memory requirement issue exists in many assemblers with which we compared in this study. As shown in Table 3, if a genome can not be assembled by Clover, the genome has the high probability that it can not be assembled by other assemblers we used in this study.

### Future works

We leave the parallelization of program as a future work that will further improve the performance of Clover. Besides, we leave the exploration of other possible clustering algorithms to further improve Clover as another future work.

## Conclusions

In this study, we developed a new clustering-oriented de novo assembler, called Clover, that integrates the flexibility of the overlap-layout-consensus approach on clustering *k*-mers and the efficiency of the de Bruijn graph method, with which we improve the robustness with respect to sequencing error especially using large *k*-mers. We discovered the effect of our clustering approach that not only improves the assembly result but also accelerates the assembly process by simplifying analysis on the *Leptospira shermani* assembly. The evaluation of Clover on GAGE datasets finally shows that it achieves a superior assembly quality in terms of corrected N50 and E-size while remaining a significantly competitive in run time.

## Availability and requirements

*Project name* Clover.

*Project home page* https://oz.nthu.edu.tw/~d9562563/

*Operating system(s)* Linux.

*Programming language* C, Python and Cython.

*Other requirements* Python-devel to develop Python extensions.

*License* GNU GPL2.

*Any restrictions to use by non-academics* None.

## Supplementary information

Additional file 1 Clover source code for Linux. Please refer to ‘Installation of Clover’.

Additional file 2 Datasets and Installation of Clover. GAGE dataset list and locations, and build Clover’s executing and programming environments.

Additional file 3 Section S1—*Leptospira shermani* assembly statistics results. Section S2—Clover assembly statistics results. Clover output screen text.

Additional file 4 Table S1—Memory requirements (GB) of k versus p correlation on *Leptospira shermani* assembly. Table S2—Sensitivity comparison of different minimum overlapping lengths on *Rhodobacter sphaeroides* assembly. Supplemental analysis of Clover.

## Data Availability

The evaluation datasets used during the current study are available in the https://gage.cbcb.umd.edu/data/. The source code of Clover is available at https://oz.nthu.edu.tw/~d9562563/src.html.

## Abbreviations

ABySS: Assembly by short sequences
bp: Base pairs
CABOG: Celera assembler with the best overlap graph
Clover: Clustering-oriented de novo assembler
DNA: Deoxyribonucleic acid
E-size: Expected size
GAGE: Genome assembly gold-standard evaluation
GC-content: Guanine-cytosine content
kb: Kilo base pairs
Mb: Mega base pairs
MSR-CA: Maryland super-read celera assembler
NGS: Next-generation sequencing
PCR: Polymerase chain reaction
rRNA: Ribosomal ribonucleic acid
SGA: String graphs assembler
SOAPdenovo: Short oligonucleotide analysis package de novo
SPAdes: St. Petersburg genome assembler
tRNA: Transfer ribonucleic acid
