# Supplementary material for: Clover: a clustering-oriented de novo assembler for Illumina sequences
Source: BMC Bioinformatics. 2020 Nov 17;21:528. doi: 10.1186/s12859-020-03788-9 (PMC7672897; doi:10.1186/s12859-020-03788-9)
Supplement: Supplementary file 2 — Datasets and Installation of Clover. GAGE dataset list and locations, and build Clover’s executing and programming environments. [file 12859_2020_3788_MOESM2_ESM.docx]

**Additional File 2**

## Datasets

To evaluate the assembly correctness of Clover, we have tested three typical datasets in the GAGE study: *Staphylococcus aureus* (2.9 Mb), *Rhodobacter sphaeroides* (4.6 Mb) and human chromosome 14 (88.3 Mb). Each dataset has original reads, Quake corrected reads and Allpaths-LG corrected reads. The result with the best scaffold N50 on these three datasets is selected for assembly comparison in Table 2.

Each dataset in the GAGE study is available at http://gage.cbcb.umd.edu/data/<genome>/, where <genome> is *Staphylococcus_aureus*, *Rhodobacter_sphaeroides* and Hg_chr14, respectively, and consists of the following files:

- Original reads are in Data.original.tgz archive; Quake corrected reads are in Data.quakeCor.tgz archive; Allpaths-LG corrected reads are in Data.allpathsCor.tgz archive; each archive includes frag_{1,2}.fastq, shortjump_{1,2}.fastq and, when available, longjump_{1,2}.fastq.
- GAGE final assemblies are in Assembly.tgz archive.
- Reference genomic sequence file is genome.fasta, which is located in the Data.original/ directory.

## Installation of Clover

1. Download Clover package and unpack it to your target folder.

$ tar -zxvf clover-x.x.tar.gz.

1. For testing Clover, test data is available at ‘Test Case’ of our website, and the help command will show you all parameters syntax and their defaults.

$ cd clover-x.x.

$ clover –h.

1. For programming, build Clover executable code from src folder’s source files.

- kp1.c is compiled to kpgraph.so:

$ python kp2.py build.

$ mv build/lib.linux-x86_64-2.7/kpgraph.so ./.

- cython compiles clover.py to clover.c:

$ cython --embed clover.py.

- gcc compiles clover.c to the executable code clover:

$ gcc -I/usr/include/python2.7 -o clover clover.c -lpython2.7 -lpthread -lm -lutil -ldl.
