# Supplementary material for: Clover: a clustering-oriented de novo assembler for Illumina sequences
Source: BMC Bioinformatics. 2020 Nov 17;21:528. doi: 10.1186/s12859-020-03788-9 (PMC7672897; doi:10.1186/s12859-020-03788-9)
Supplement: Supplementary file 3 — Section S1—Leptospira shermani assembly statistics results. Section S2—Clover assembly statistics results. Clover output screen text. [file 12859_2020_3788_MOESM3_ESM.docx]

**Additional File 3**

**Section S1 – *Leptospira shermani* assembly statistics results**

(a) –p: 0

| *start:*  *Input reads, reads: 7062546*  *K-mers in reads: 247189110*  *K-mers in k-mer set: 33692986*  *Nodes to build the de Bruijn graph: 33692986*  *Finish the first step.*  *nodes: 33692986*  *paths: 2905641*  *Trim low-frequency edges.*  *nodes: 24977213*  *paths: 2657988*  *Prune tips, bubbles, and erroneous connections.*  *nodes: 4201001*  *paths: 87234*  *Iteratively relink graph with shorter k-mer.*  *nodes: 3720535*  *paths: 6164*  *Finish clean graph.*  *Contig set:*  *nodes: 3619862*  *total length: 3817592*  *contigs: 5070*  *max length: 8711*  *average length 752*  *n50: 958*  *Run a scaffolding*  *Scaffold set:*  *nodes: 3939934*  *total length: 3947032*  *contigs: 182*  *max length: 178311*  *average length 21686*  *n50: 54174*  *end:* |
| --- |

* The assembly results are indicated in bold.

(b) –p: 1

| *start:*  *Input reads, reads: 7062546*  *K-mers in reads: 247189110*  *K-mers in k-mer set: 33692986*  *Components of k-mers in k-mer set: 15874827*  *Split the nodes.*  *Nodes to build the de Bruijn graph: 15969082*  *Finish the first step.*  *nodes: 15969082*  *paths: 782493*  *Trim low-frequency edges.*  *nodes: 3968762*  *paths: 20218*  *Prune tips, bubbles, and erroneous connections.*  *nodes: 3910665*  *paths: 15691*  *Iteratively relink graph with shorter k-mer.*  *nodes: 3858620*  *paths: 1767*  *Finish clean graph.*  *Contig set:*  *nodes: 3827964*  *total length: 3874803*  *contigs: 1201*  *max length: 34164*  *average length 3226*  *n50: 5471*  *Run a scaffolding*  *Scaffold set:*  *nodes: 3892400*  *total length: 3896963*  *contigs: 117*  *max length: 195969*  *average length 33307*  *n50: 85322*  *end:* |
| --- |

(c) –p: 2

| *start:*  *Input reads, reads: 7062546*  *K-mers in reads: 247189110*  *K-mers in k-mer set: 33692986*  *Components of k-mers in k-mer set: 11792400*  *Split the nodes.*  *Nodes to build the de Bruijn graph: 11917810*  *Finish the first step.*  *nodes: 11917810*  *paths: 532633*  *Trim low-frequency edges.*  *nodes: 3957814*  *paths: 17946*  *Prune tips, bubbles, and erroneous connections.*  *nodes: 3907569*  *paths: 14674*  *Iteratively relink graph with shorter k-mer.*  *nodes: 3859836*  *paths: 2558*  *Finish clean graph.*  *Contig set:*  *nodes: 3816087*  *total length: 3859104*  *contigs: 1103*  *max length: 26780*  *average length 3498*  *n50: 6081*  *Run a scaffolding*  *Scaffold set:*  *nodes: 3875467*  *total length: 3880030*  *contigs: 117*  *max length: 145282*  *average length 33162*  *n50: 63316*  *end:* |
| --- |

**Section S2 - Clover assembly statistics results**

(a) *Staphylococcus aureus*

| *start:*  *Input reads, reads: 1799750*  *K-mers in reads: 121151730*  *K-mers in k-mer set: 2860555*  *Nodes to build the de Bruijn graph: 2860555*  *Finish the first step.*  *nodes: 2860555*  *paths: 2508*  *Trim low-frequency edges.*  *nodes: 2860603*  *paths: 2084*  *Prune tips, bubbles, and erroneous connections.*  *nodes: 2847585*  *paths: 1052*  *Iteratively relink graph with shorter k-mer.*  *nodes: 2843148*  *paths: 401*  *Finish clean graph.*  *Contig set:*  *nodes: 2808512*  *total length: 2812480*  *contigs: 128*  *max length: 142590*  *average length 21972*  *n50: 45776*  *Run a scaffolding.*  *Scaffold set: 1*  *nodes: 2808512*  *total length: 2812480*  *contigs: 128*  *max length: 142590*  *average length 21972*  *n50: 45776*  *Run a scaffolding.*  *Scaffold set: 2*  *nodes: 2860484*  *total length: 2860856*  *contigs: 12*  *max length: 1490045*  *average length 238404*  *n50: 1490045*  *end:* |
| --- |

* The results of Num and N50 are indicated in bold.

(b) *Rhodobacter sphaeroides*

| *start:*  *Input reads, reads: 2722066*  *K-mers in reads: 152435625*  *K-mers in k-mer set: 4554148*  *Nodes to build the de Bruijn graph: 4554148*  *Finish the first step.*  *nodes: 4554148*  *paths: 2344*  *Trim low-frequency edges.*  *nodes: 4552253*  *paths: 1603*  *Prune tips, bubbles, and erroneous connections.*  *nodes: 4528817*  *paths: 832*  *Iteratively relink graph with shorter k-mer.*  *nodes: 4525151*  *paths: 487*  *Finish clean graph.*  *Contig set:*  *nodes: 4523157*  *total length: 4543542*  *contigs: 453*  *max length: 88519*  *average length 10029*  *n50: 20413*  *Run a scaffolding.*  *Scaffold set: 1*  *nodes: 4523157*  *total length: 4542417*  *contigs: 428*  *max length: 88519*  *average length 10613*  *n50: 21217*  *Run a scaffolding.*  *Scaffold set: 2*  *nodes: 4636413*  *total length: 4639068*  *contigs: 59*  *max length: 2482925*  *average length 78628*  *n50: 2482925*  *end:* |
| --- |

(c) Human chromosome 14

| *start:*  *Input reads, reads: 45711162*  *K-mers in reads: 948431974*  *K-mers in k-mer set: 95365346*  *Components of k-mers in k-mer set: 83236602*  *Split the nodes.*  *Nodes to build the de Bruijn graph: 88139675*  *Finish the first step.*  *nodes: 88139675*  *paths: 788554*  *Trim low-frequency edges.*  *nodes: 85966174*  *paths: 303749*  *Prune tips, bubbles, and erroneous connections.*  *nodes: 84910377*  *paths: 262348*  *Iteratively relink graph with shorter k-mer.*  *nodes: 85457167*  *paths: 83254*  *Finish clean graph.*  *Contig set:*  *nodes: 74124604*  *total length: 76062237*  *contigs: 24527*  *max length: 60596*  *average length 3101*  *n50: 4123*  *Run a scaffolding.*  *Scaffold set: 1*  *nodes: 74188364*  *total length: 76021243*  *contigs: 23201*  *max length: 60596*  *average length 3276*  *n50: 4478*  *Run a scaffolding.*  *Scaffold set: 2*  *nodes: 84501702*  *total length: 84742020*  *contigs: 3042*  *max length: 654253*  *average length 27857*  *n50: 103975*  *Run a scaffolding.*  *Scaffold set: 3*  *nodes: 91117569*  *total length: 91282600*  *contigs: 2089*  *max length: 3060417*  *average length 43696*  *n50: 826039*  *end:* |
| --- |
