# Supplementary material for: Clover: a clustering-oriented de novo assembler for Illumina sequences
Source: BMC Bioinformatics. 2020 Nov 17;21:528. doi: 10.1186/s12859-020-03788-9 (PMC7672897; doi:10.1186/s12859-020-03788-9)
Supplement: Supplementary file 4 — Table S1—Memory requirements (GB) of k versus p correlation on Leptospira shermani assembly. Table S2—Sensitivity comparison of different minimum overlapping lengths on Rhodobacter sphaeroides assembly. Supplemental analysis of Clover. [file 12859_2020_3788_MOESM4_ESM.doc]

# Additional file 4

**Table S1 – Memory requirements (GB) of k versus p correlation on *Leptospira shermani* assembly**

| *k* *p* | 0 | 1 | 2 | 3 |
| --- | --- | --- | --- | --- |
| 25 | 18.6 | 10.1 | 10.1 | 10.2 |
| 28 | 18.5 | 16.8 | 12.8 | 12.8 |
| 31 | 18.4 | 16.7 | 9.4 | 9.8 |
| 34 | 18.2 | 16.4 | 12.1 | 12.1 |
| 37 | 18.0 | 16.2 | 9.5 | 9.4 |
| 40 | 17.7 | 16.2 | 11.7 | 11.5 |
| 43 | 17.4 | 15.7 | 20.3 | 9.1 |
| 46 | 17.0 | 15.6 | 20.0 | 10.7 |
| 49 | 16.7 | 15.2 | 19.6 | 9.3 |
| 52 | 16.2 | 14.9 | 19.3 | 11.9 |
| 55 | 15.8 | 14.7 | 18.9 | 11.5 |
| 58 | 15.2 | 14.4 | 18.5 | 23.0 |
| 61 | 14.7 | 14.1 | 17.7 | 22.5 |
| 64 | 14.0 | 13.7 | 17.5 | 21.6 |

*k*, the length of *k*-mers; *p*, the level of error allowance on the *k*-mers.

**Table S2 - Sensitivity comparison of different minimum overlapping lengths on *Rhodobacter sphaeroides* assembly**

| *m* |  |  | Contigs |  |  |  |  |  | Scaffolds |  |  |  |
| --- | --- | --- | --- | --- | --- | --- | --- | --- | --- | --- | --- | --- |
|  | *Num* | *N50* | *E-size* | *Errs* | *N50C* | *E-sizeC* | *Num* | *N50* | *E-size* | *Errs* | *N50C* | *E-sizeC* |
|  |  | (kb) | (kb) |  | (kb) | (kb) |  | (kb) | (kb) |  | (kb) | (kb) |
| 12 | **402** | **23.5** | **27.2** | 20 | **21.8** | **24.8** | **57** | 1277 | 1126 | **1** | 1277 | 1126 |
| 15 | 419 | 22.6 | 25.8 | 20 | 20.5 | 23.8 | **57** | 1278 | 1126 | **1** | 1278 | 1126 |
| 18 | 432 | 22.1 | 25.1 | 20 | 19.9 | 23.2 | 58 | 1277 | 1126 | **1** | 1277 | 1126 |
| 21 | 445 | 20.9 | 24.1 | 20 | 19.6 | 22.1 | 58 | **2483** | **1795** | **1** | **2483** | **1795** |
| 24 | 453 | 20.1 | 23.8 | 19 | 19.5 | 21.9 | 59 | **2483** | **1795** | **1** | **2483** | **1795** |
| 27 | 464 | 19.9 | 23.5 | 19 | 19.1 | 21.6 | 60 | 1421 | 1168 | **1** | 1421 | 1168 |
| 30 | 479 | 19.6 | 22.3 | 17 | 18.3 | 21.0 | 61 | 1421 | 1168 | **1** | 1421 | 1168 |
| 33 | 497 | 19.1 | 21.6 | 17 | 17.6 | 20.3 | 62 | 1421 | 1168 | **1** | 1421 | 1168 |
| 36 | 512 | 17.7 | 20.3 | 17 | 16.9 | 19.2 | 62 | 1421 | 1168 | **1** | 1421 | 1168 |
| 39 | 526 | 17.3 | 19.9 | 17 | 15.8 | 18.8 | 66 | 1421 | 1081 | **1** | 1421 | 1081 |
| 42 | 538 | 16.9 | 19.4 | 17 | 15.2 | 18.3 | 66 | 1421 | 1081 | **1** | 1421 | 1081 |
| 45 | 555 | 15.2 | 18.9 | **16** | 14.0 | 17.7 | 67 | 1421 | 1081 | **1** | 1421 | 1081 |

*m*, the length of minimum overlapping; *Num*, the number of sequences produced; *N50*, the N50 statistic calculated with respect to the total length of sequences produced; *E-size*, the most likely size of the sequence containing some random base in the genome; *Errs*, the number of misjoins and for the contig value, also the number of indels >5 bases; *N50C*, the N50 calculated after splitting all sequences at error locations; and *E-sizeC*, the E-size calculated after splitting all sequences at error locations. The best result in each column is indicated in bold.
